# Sonographic Characteristics of Fistulous Lesions in Facial Acne: A Comprehensive Analysis

**DOI:** 10.1111/jocd.70478

**Published:** 2025-09-29

**Authors:** Yufang You, Wenqing Ran, Yimin Su, Bibo Li, Shigen Zhong

**Affiliations:** ^1^ Department of Ultrasound, Chongqing General Hospital Chongqing University Chongqing China; ^2^ Department of Oncology, Chongqing General Hospital Chongqing University Chongqing China; ^3^ Department of Dermatology Shanghai Sixth People's Hospital Shanghai China

**Keywords:** facial acne, fistulous lesion, high‐frequency ultrasound, morphological analysis, skin inflammation

## Abstract

**Objective:**

This study aimed to investigate the sonographic characteristics of fistulous lesions in facial acne.

**Methods:**

Facial acne patients were classified based on clinical grading criteria, with or without fistulous lesions, and subsequently underwent high‐frequency ultrasound assessment to evaluate lesion characteristics. Statistical analyses were conducted to compare clinical and sonographic features between fistula and nonfistula groups.

**Results:**

A total of 2355 acne lesions in 189 patients were studied, and 343 fistulous lesions were detected in 71 patients (fistula group). The male proportion and the age of the fistula group were higher than those of the nonfistula group (50.70% vs. 30.51%; 23.21 vs. 26.72, respectively). Among the fistula group, a strong positive correlation was found between the highest grade of fistulous lesions per patient and the highest grade of each patient (*r*_s_ = 0.853, *p* < 0.001). In the fistula group (totaling 909 lesions), the length of fistulous lesions was significantly greater than that of nonfistulous lesions (*F* = 251.339, *p* < 0.001). Across all 189 patients, dermal thickness, lesion depth, lesion length, and lesion area were significantly greater in the fistula group compared to the nonfistula group (dermal thickness: *t* = 19.582, *p* < 0.001; depth: *t* = 7.613, *p* < 0.001; length: *t* = 21.169, *p* < 0.001; lesion area: *z* = −20.580, *p* < 0.001, respectively). High‐frequency ultrasound revealed thickened dermal layers in acne lesions, with fistulas present across various skin layers, including the epidermis, dermis, and subcutaneous tissue. Fistulous lesions typically presented as low banded pattern echoes (length‐to‐depth ratio ≥ 3:1), with blood flow signals varying according to inflammatory activity.

**Conclusion:**

High‐frequency ultrasound effectively characterizes fistulous lesions in facial acne, providing critical insights for clinical management and further clinical research.

## 1 Introduction

Acne vulgaris, a prevalent inflammatory disorder of the pilosebaceous unit, predominantly affects the facial and truncal regions, with a peak incidence during adolescence (12–24 years old), affecting approximately 85% of this demographic [1, 2]. Epidemiological data from Chinese population studies indicate an acne prevalence rate of 8.1% [3]. The etiopathogenesis of acne remains incompletely elucidated, though current evidence suggests a multifactorial process involving genetic predisposition, hormonal stimulation of sebaceous gland hyperactivity, follicular hyperkeratinization, colonization by *Propionibacterium acnes* , and subsequent inflammatory and immune responses [4].

The clinical manifestations of acne progress through distinct pathological stages. Superficial dermal inflammation manifests as papules or pustules, while deeper dermal involvement leads to cystic or nodular formations. The resolution phase of these lesions is frequently complicated by aberrant collagen metabolism, resulting in various scar types, including atrophic scars (80%–90% of cases), hypertrophic scars, and keloids [5]. A prospective cohort study demonstrated that 43% (843/1972) of acne patients developed scarring, with significant implications for their psychosocial well‐being and quality of life [6].

Accurate acne classification serves as the cornerstone for therapeutic decision‐making and outcome assessment. The Chinese clinical guidelines adopt a lesion‐based classification system, categorizing acne into three types and four grades [7]. This classification system informs the therapeutic approach, with topical agents constituting first‐line treatment for mild to moderate cases, while moderate to severe presentations often require adjunctive systemic therapy [8]. However, therapeutic challenges persist, with treatment failure rates reaching 52% [9], potentially attributable to the inability to detect subclinical or deep‐seated lesions through conventional clinical examination.

The emergence of high‐frequency ultrasound as a diagnostic modality offers new insights into acne pathology. Previous ultrasonographic studies have identified characteristic abnormalities including pseudocysts, folliculitis, fistulous tracts, and calcinosis [10]. While pseudocysts, folliculitis, and nodules demonstrate clear clinical‐pathological correlations, the detection and characterization of fistulous lesions remain heavily dependent on sonographic evaluation. This diagnostic gap underscores the need for focused investigation, particularly regarding the correlation between fistulous lesions and clinical acne severity, their pathogenic mechanisms, and their role in disease progression. Current literature is limited, with only 19 fistulous lesions reported across five patients in existing studies [10].

This study aims to advance our understanding of fistulous lesions in facial acne through detailed sonographic characterization and comparative analysis with nonfistulous lesions. The findings are expected to provide valuable data for optimizing therapeutic strategies and informing future clinical research directions in acne management.

## 2 Materials and Methods

### 2.1 Study Design

The research focused on patients diagnosed with facial acne vulgaris by a senior dermatologist, who also underwent sonographic examinations conducted by a senior ultrasound physician. The study period spanned from September 2021 to September 2024.

Patients were enrolled based on the following inclusion criteria: (1) age 16 years or older, (2) clinical diagnosis and sonographic examination of the same facial acne lesion, and (3) sonographic examination performed using color Doppler ultrasound. Exclusion criteria included: (1) patients who did not consent to the evaluation of clinical classification or sonographic examination, and (2) those with concomitant systemic or regional cutaneous diseases affecting the same or adjacent areas as the acne.

The number of facial acne lesions per patient ranged from 3 to 40. A senior dermatologist clinically graded each lesion according to the China Clinical Classification of Acne [7] (Table 1), with the highest‐grade lesion determining the patient's final diagnostic grade.

**TABLE 1:** China clinical classification of acne vulgaris.

Severity	Grade	Clinical feature
Mild	Grade I	Comedones
Moderate	Grade II	Involves inflammatory red papules
	Grade III	Involves pustules
Severe	Grade IV	Involves nodules and/or cysts

### 2.2 Ultrasound Protocol

The ultrasound equipment utilized in this study was the MYLABONE Ultrasound Diagnostic System (Esaote Europe B.V.). Settings were optimized with the lowest pulse repetition frequencies, minimal wall filters, and color gain adjusted just below the noise threshold. The compact linear ultrasound probes operated at upper frequencies of 25 MHz, and sonograms were recorded in 2‐dimensional grayscale and color Doppler modes.

High‐frequency sonographic examinations were conducted by the same senior ultrasound physician, following a standardized protocol. Patients were positioned supine, and a generous amount of ultrasound gel was applied to the skin surface in the lesion area. Ultrasound examination was performed sequentially, starting with the frontal and temple regions, followed by the bilateral cheeks, lips, and finally the chin. Both transverse and longitudinal sweeps were conducted in each region, moving systematically from right to left and from top to bottom. The ultrasound probe was carefully positioned over the predominant lesions.

For each lesion, morphological characteristics were documented, including dermal thickness, the longest diameter in both depth and length, local blood flow, and the presence of fistulas. In sonographic examinations, fistulous lesions were defined as well‐demarcated, band‐like anechoic or hypoechoic structures [10].

### 2.3 Statistical Analysis

This study categorized lesions into two distinct types based on ultrasonographic characteristics: (1) fistulous lesions (demonstrating typical fistula morphology on ultrasound imaging) and (2) nonfistulous lesions (presenting alternative sonographic features including pseudocysts, folliculitis, or calcinosis). Based on these classifications, patients were stratified into two groups: (1) the fistula group (patients exhibiting ≥ 1 fistulous lesion) and (2) the nonfistula group (patients showing complete absence of fistulous lesions).

Data analysis was performed using SAS 9.3 software. In addition to the general descriptive analysis of the data, the following statistical tests were listed in Table 2. The significance level was set at 5% (95% confidence interval; *p* < 0.05).

**TABLE 2:** A summary of variable and statistical test.

Variable	Statistical test
Number of fistulas in male and female patients	Wilcoxon rank sum test
Area of fistulous lesions and nonfistulous lesions	
Area of lesions in fistula group and nonfistula group	
Patient age and the number of fistulas	Spearman rank correlation analysis
Highest grade of fistulous lesions per patient and the highest grade of patient in fistula group	
Proportion of male patients in the fistula group and nonfistula group	Chi‐square test
Mean age of fistula group and nonfistula group	*t*‐test
Dermal thickness, depth, and length of lesions in fistula group and nonfistula group	
Dermal thickness, lesion depth, the length of fistulous and nonfistulous lesions	GLMM

## 3 Results

### 3.1 Demographic and Clinical Feature

The baseline of facial acne patients and clinical grading of lesions was shown in Tables 3 and 4, respectively. Sonographic evaluations were conducted on 189 patients, encompassing a total of 2356 facial acne lesions. The study population comprised 38.1% males and 61.9% females, with a mean age of 24.5 ± 4.9 years.

**TABLE 3:** Demographic characteristics of patients.

Characteristics	Total patients	Fistula group	Nonfistula group
Number of patients	189	71	118
Gender			
Female	117	35	82
Male	72	36	36

**TABLE 4:** Clinical grading of lesions.

Classification	Total lesions (*n* = 2356)	Fistula group		Nonfistula group
		Fistulous lesions (*n* = 343)	Nonfistulous lesions (*n* = 566)	Nonfistulous lesions (*n* = 1447)
Grade I	870	0	126	744
Grade II	774	147	198	429
Grade III	548	191	152	205
Grade IV	164	5	90	69

Among the evaluated total lesions, 870 were graded as grade I, 774 as grade II, 548 as grade III, and 164 as grade IV. Of the 189 patients evaluated, 71 (37.6%) presented with fistulous lesions, comprising 343 individual lesions. These lesions demonstrated the following severity distribution: Grade II (147 lesions, 42.9%), Grade III (191 lesions, 55.7%), and Grade IV (5 lesions, 1.5%). Collectively, fistulous lesions represented 14.6% (343/2356) of all documented lesions.

### 3.2 Differences of Number of Fistulas in Gender and Age

In the fistula group, both male and female patients had a mean of 5 fistulas. The Wilcoxon rank sum test for two independent samples revealed no statistically significant difference in the number of fistulas between male and female patients (*z* = −0.299, *p* = 0.765). Additionally, Spearman rank correlation analysis indicated no significant correlation between patient age and the number of fistulas (*r*_s_ = −0.033, *p* = 0.784). These results are summarized in Table 5.

**TABLE 5:** The number of fistulas in male and female patients.

	Male (*n* = 36)	Female (*n* = 35)	*z*	*p*
Mean number of fistulas	5.00 (3.25–6.00)	5.00 (3.00–6.00)	−0.299	0.765

### 3.3 Gender and Age Differences Between Fistula and Nonfistula Group

As shown in Table 6, both the fistula and nonfistula groups included 36 male patients each. However, the fistula group demonstrated a younger mean age (23.21 years) relative to the nonfistula group (26.72 years).

**TABLE 6:** Demographic characteristics of fistula group and nonfistula group.

	Fistula group (*n* = 71)	Nonfistula group (*n* = 118)	*t*/*χ* ^2^	*p*
Male	36 (50.70)	36 (30.51)	7.667	0.006
Age (years)	26.72 ± 4.36	23.21 ± 4.83	−5.013	< 0.001

The chi‐square test demonstrated a significantly higher proportion of male patients in the fistula group compared to the nonfistula group (*χ* ^2^ = 7.667, *p* = 0.006). Furthermore, independent samples *t*‐tests indicated that the mean age of the fistula group was higher than that of the nonfistula group (*t* = −5.013, *p* < 0.001).

### 3.4 Highest Grade of Fistulous Lesions and the Highest Grade of Fistula Patient

As previously described, each lesion was clinically graded by dermatologists according to its characteristics, with the highest‐grade lesion determining the patient's final diagnostic grade. Among the fistula group (*n* = 71), the highest grade distribution was as follows: 22 patients were classified as grade III, and 49 patients were classified as grade IV. Regarding the highest grade of fistulous lesions per patient (each patient may contain one or more fistulous lesions), the distribution was as follows: 24 lesions were grade II, 45 lesions were grade III, and 2 lesions were grade IV.

Spearman rank correlation analysis revealed a strong positive correlation between the highest grade of fistulous lesions per patient and the highest grade of patients in the fistula group (*r*_s_ = 0.853, *p* < 0.001). These results are summarized in Table 7.

**TABLE 7:** Highest grade of fistulous lesions and the highest grade of fistula patients.

Highest grade of fistulous lesions	Highest grade of fistula patient		*r*_s_	*p*
	Grade III	Grade IV		
GradeII	21	3	0.853	< 0.001
Grade III	1	44		
Grade IV	0	2		

### 3.5 Sonographic Characteristics of Fistulous and Nonfistulous Lesions in Fistula Group

A total of 343 fistulous lesions and 566 nonfistulous lesions were identified among the fistula group. The findings from the high‐frequency ultrasound examinations are summarized in Table 8. Based on the generalized linear mixed model analysis, no statistically significant differences were observed in dermal thickness or lesion depth between fistulous and nonfistulous lesions (dermal thickness: *F* = 0.939, *p* = 0.333; lesion depth: *F* = 1.364, *p* = 0.243). However, the length of fistulous lesions was significantly greater than that of nonfistulous lesions (*F* = 251.339, *p* < 0.001). Additionally, the stratified Wilcoxon rank sum test revealed that the area of fistulous lesions was significantly larger than that of nonfistulous lesions (*z* = −15.504, *p* < 0.001).

**TABLE 8:** Sonographic parameters of lesions in fistula group (mean ± SD).

Characteristics	Fistulous lesions (*n* = 343)	Nonfistulous lesions (*n* = 566)	*F*/*z*	*p*
Dermal thickness (mm)	1.78 ± 0.28	1.73 ± 0.38	0.939	0.333
Depth (mm)	2.59 ± 0.52	2.65 ± 1.37	1.364	0.243
Length (mm)	8.75 ± 1.57	5.21 ± 3.61	251.339	< 0.001
Area (mm^2^)	18.17 (12.97–25.01)	7.43 (4.25–11.88)	−15.504	< 0.001

### 3.6 Sonographic Characteristics of Lesions in Fistula Group and Nonfistula Group

The study identified 909 lesions in the fistula group and 1447 lesions in the nonfistula group. The findings from the high‐frequency ultrasound examinations are presented in Table 9. Independent samples *t*‐tests revealed that the dermal thickness, depth, and length of lesions in the fistula group were significantly greater than those in the nonfistula group (dermal thickness: *t* = 19.582, *p* < 0.001; lesion depth: *t* = 7.613, *p* < 0.001; lesion length: *t* = 21.169, *p* < 0.001). Furthermore, the Wilcoxon rank sum test for two independent samples demonstrated that the area of lesions in the fistula group was significantly larger than that in the nonfistula group (z = −20.580, *p* < 0.001).

**TABLE 9:** Sonographic parameters of lesions in fistula group and nonfistula group (mean ± SD).

Characteristics	Fistula group (lesions *n* = 909)	Nonfistula group (lesions *n* = 1447)	*t*/*z*	*p*
Dermal thickness (mm)	1.75 ± 0.35	1.49 ± 0.23	19.582	< 0.001
Depth (mm)	2.63 ± 1.12	2.27 ± 1.13	7.613	< 0.001
Length (mm)	6.55 ± 3.46	3.83 ± 2.19	21.169	< 0.001
Area (mm^2^)	11.11 (6.22–19.22)	5.02 (2.76–7.64)	−20.580	< 0.001

### 3.7 Sonographic Characteristics of Fistulous Lesions

**FIGURE 1:**
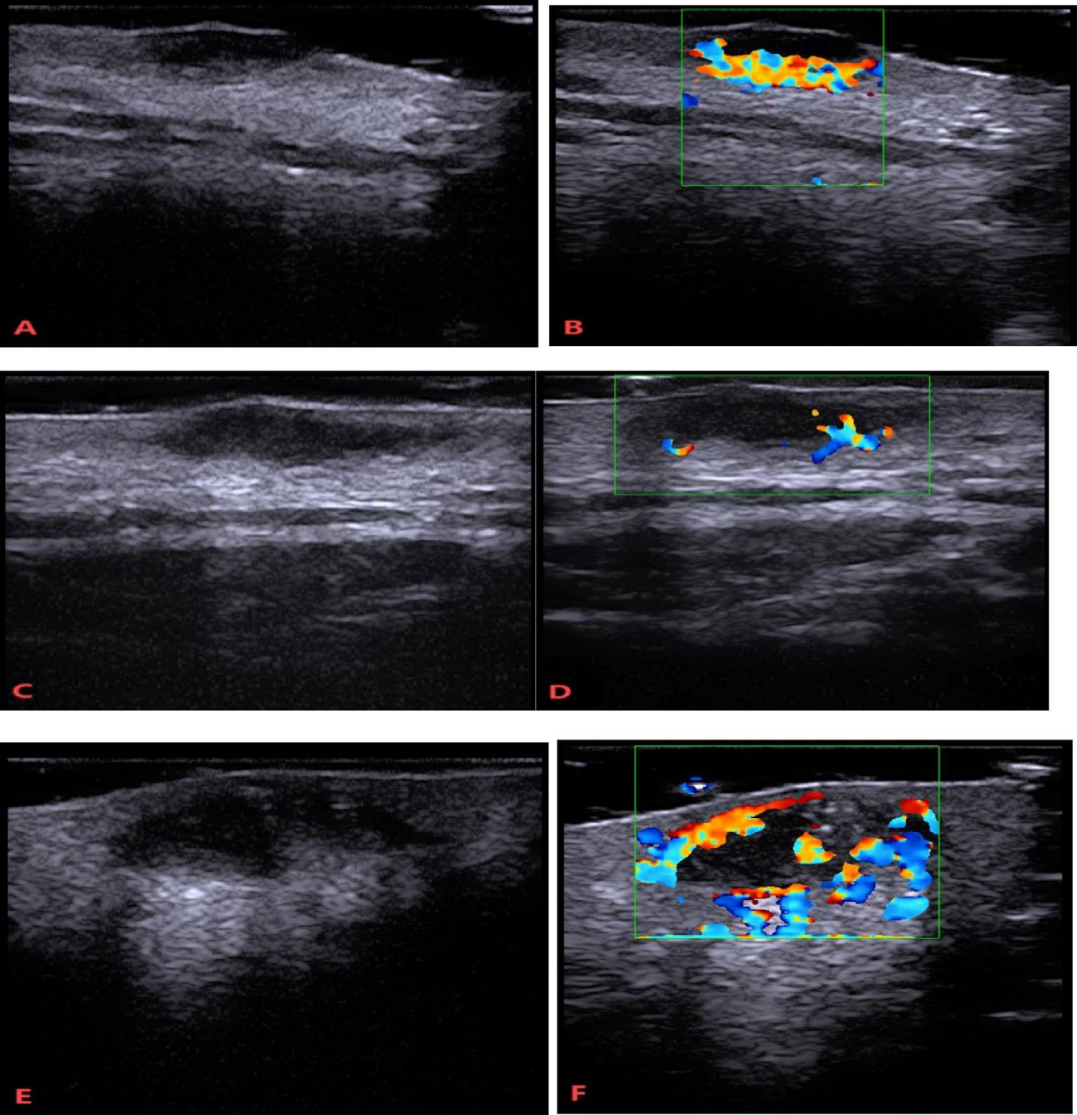
Representative sonographic images of fistulous lesions (A–F).

The morphological characteristics of facial acne with fistulous lesions, as visualized by high‐frequency ultrasound, are depicted in the following figures (Figure 1).

Figure 1A illustrates a fistulous lesion localized within both the epidermal and dermal layers, accompanied by partial loss of epidermal keratinization. The lesion presented with a slightly raised surface, indistinct borders, and an irregular shape and base. Abundant blood flow was observed within the lesion, Figure 1B. Based on its clinical features, the acne with fistulous lesions was classified as grade II by the dermatologist.

Figure 1C depicts a fistulous lesion confined to the dermal layer, with mild epidermal keratinization. The surface appeared relatively regular, while the boundaries remained indistinct. The lesion displayed a creeping morphology with an irregular base. Blood flow signals and nourishing vessels were detectable within the lesion Figure 1D. This acne with fistulous lesions was categorized as grade III according to dermatological assessment.

Figure 1E shows a fistulous lesion extending into both the dermal and subcutaneous tissue layers, with mild epidermal hyperkeratosis. The surface maintained relative regularity, while the overall shape and base exhibited irregularity, including downward irregular elevation. Internal hyperechoic foci suggestive of calcinosis were observed. The lesion demonstrated abundant blood flow (Figure 1F). This acne with fistulous lesions was graded as IV by the dermatologist.

Collectively, Figure 1A–F highlights several key ultrasonographic characteristics: thickening of the dermal layer at the acne lesion site, potential involvement of multiple skin layers (epidermis, dermis, and subcutaneous tissue) by fistulous tracts, and the characteristic presentation of fistulous lesions as hypoechoic band‐like structures. The intensity of blood flow signals correlated with the degree of inflammatory activity within the lesions.

## 4 Discussion and Conclusion

Acne clinical classification serves as a crucial foundation for both treatment strategies and efficacy evaluation. However, current classification methods, whether the internationally modified approach based on lesion count [11, 12] or the Chinese clinical classification emphasizing lesion nature [7], remain superficial in their assessments. Acne lesions often extend deep into the skin, sometimes reaching the hypodermis, which complicates accurate diagnosis. While serial histologic diagnosis is considered the gold standard for skin lesions, its routine application is hindered by the risk of facial scarring. Misjudgments in clinical evaluations can lead to treatment failures. Consequently, sonography has emerged as the preferred noninvasive diagnostic tool for acne, offering precise anatomical data such as lesion depth and length, local blood flow, and the presence of specific structures. These pathophysiological insights are invaluable for comprehensive acne management.

Because of these advantages, high‐frequency ultrasound has become a widely utilized tool for the evaluation and differentiation of acne. In a prospective study involving 70 patients with moderate‐to‐severe acne vulgaris, both clinical assessments and a sonographic scoring system were employed [13]. The results demonstrated consistency between clinical and high‐frequency ultrasound grades before and after treatment, and higher grades were observed with ultrasound. Similarly, another study utilized Color Doppler Ultrasound to evaluate papulopustular rosacea management [14]. While clinical improvements were noted posttreatment, no significant ultrasonographic changes were detected, underscoring the limitations of clinical evaluations alone in assessing the inflammatory process. Furthermore, high‐frequency and ultra‐high‐frequency ultrasound have proven effective in distinguishing severe acne vulgaris from hidradenitis suppurativa, a differentiation that is challenging clinically [15].

Despite acne's global prevalence, research on ultrasound imaging of facial acne vulgaris with fistulous lesions remains limited. A prior study of 245 acne lesions in 20 patients identified sonographic abnormalities including pseudocysts, folliculitis, fistulas, and calcinosis [10]. While pseudocysts, folliculitis, and nodules are relatively easy for dermatologists to identify, fistulas often evade clinical detection. Notably, fistulous tracts have been reported to cause nearly facial disfigurement in one case [16], highlighting the need for precise diagnosis and complex treatment. A limitation of previous studies is the small sample size. For instance, only 19 fistulous lesions were detected in 5 patients, with 13 traversing both the dermis and upper hypodermis and 6 confined to the dermis [10]. None of these were identified through clinical examination alone.

Our study represents the first large‐scale assessment of facial acne with fistulous lesions using both sonographic and clinical methods. We examined 2356 acne lesions in 189 patients, finding that 37.57% (71/189) of patients had fistulas—a significantly higher proportion than the 25% (5/20) reported previously. We found that the fistulous lesions accounted for 14.56% of all lesions, nearly double the earlier finding of 7.76% [10]. Additionally, the mean depth and length of the fistulous lesions were deeper but shorter compared to those reported in a previous study, where the mean maximum tract thickness was 1.59 mm and the mean major tract diameter was 14.8 mm [10], Even among the 343 fistulous lesions, we did not detect any similar thickness and diameter.

Furthermore, the fistulous lesions in the fistula group had a mean depth of 2.59 ± 0.52 mm and a mean length of 8.75 ± 1.57 mm. Based on these measurements, we recommend using a length‐to‐depth ratio of ≥ 3:1 as a criterion for characterizing fistulas.

In our study, high‐frequency ultrasound revealed thickened dermal layers in acne lesions, with fistulas present across various skin layers, including the epidermis, dermis, and subcutaneous tissue consistent with prior findings (32% in the dermis, 68% traversing both the dermis and upper hypodermis) [10].

Finally, our research findings indicated a strong positive correlation between the highest grade of fistulous lesions per patient and the highest disease severity grade within the fistula group. This raises important questions regarding how fistula severity should guide therapeutic decisions—specifically, the choice between systemic and local treatments—and how it may influence prognosis. These aspects represent a key direction for future research.

In conclusion, high‐frequency ultrasound provides a noninvasive means to evaluate the morphological changes and inflammatory responses in facial acne lesions with fistulas. Our study significantly expands the data on ultrasonic morphological characteristics of fistulous lesions of facial acne, offering valuable insights for accurate treatment and future clinical research.

## Author Contributions

All authors contributed significantly to this work. Shigen Zhong conceived and designed the study. Yufang You collected and analyzed the data. Yimi Su contributed to patient recruitment and clinical evaluation. Wenqing Ran performed the statistical analysis and interpreted the results. Bibo Li drafted the manuscript, and all authors contributed to revising it critically for important intellectual content. All authors approved the final version of the manuscript and agreed to be accountable for all aspects of the work.

## Ethics Statement

This study was approved by the Institutional Review Board of Chongqing General Hospital (Ethics Review Board No. KYD2025‐009‐01). All procedures were conducted in accordance with the ethical standards of the institutional and national research committees, as well as the 1964 Helsinki Declaration and its later amendments.

## Consent

This study was conducted as a retrospective analysis of anonymized clinical data. All patient information was de‐identified prior to analysis, and therefore, the requirement for individual informed consent was waived by the institutional review board.

## Conflicts of Interest

The authors declare no conflicts of interest.

## Data Availability

The data that support the findings of this study are available on request from the corresponding author. The data are not publicly available due to privacy or ethical restrictions.
